# Supplementary material for: Circulatory Endothelin 1-Regulating RNAs Panel: Promising Biomarkers for Non-Invasive NAFLD/NASH Diagnosis and Stratification: Clinical and Molecular Pilot Study
Source: Genes (Basel). 2021 Nov 18;12(11):1813. doi: 10.3390/genes12111813 (PMC8619934; doi:10.3390/genes12111813)
Supplement: Supplementary file 1 [file genes-12-01813-s001.zip › genes-1408340-supplementary.pdf]

Circulatory Endothelin 1- Regulating RNAs Panel: Promising Biomarkers for Non Invasive NAFLD/NASH Diagnosis and Stratification: Clinical and Molecular Pilot Study

Reda Albadawy [1,*], Sara HA. Agwa [2,*], Eman Khairy [3], Maha Saad [4], Naglaa El touchy [1], Mohamed Othman [5] ,Mohamed El Kassas[6] and Marwa Matboli [3,*]

[1] Gastroentrology, Hepatology &Infectious disease Department of, Faculty of Medicine, Benha University, Benha, Egypt; reda.albadawy@fmed.bu.edu.eg, naglaaeltoukhy@yahoo.com

[2] Clinical Pathology Department, Molecular Genomics Unit of Medical Ain Shams Research Institute , School of Medicine, Ain Shams University, Cairo, Egypt; sara.h.agwa@med.asu.edu.eg

[3] Medicinal Biochemistry and Molecular biology Department, Ain Shams University School of Medicine, Cairo, Egypt, dr_emankhairy@yahoo.com , Marwasayed472@yahoo.com , DrMarwa_Matboly@med.asu.edu.eg.

[4] Biochemistry Department, Faculty of Medicine, Modern University for Technology and Information; Cairo, Egypt, mahasaad9292@gmail.com, maha.saad@medicine.mti.edu.eg

[5] Gastroenterology and Hepatology Section Baylor College of Medicine, Houston, Texas, USA, mohamed.othman@bcm.edu

[6] Endemic Medicine and Hepato -Gastroenterology Department, Faculty of Medicine, Helwan University, m_elkassas@yahoo.com, m_elkassas@hq.helwan.edu.eg

* Correspondence: Marwa Matboli[2], Medicinal Biochemistry Department, Ain Shams University School of Medicine. Marwasayed472@yahoo.com, DrMarwa_Matboly@med.asu.edu.eg, Sara HA. Agwa, Clinical pathology, Medical Ain Shams Research institute (MASRI) Cairo, Egypt.sarakariem@gmail.com.* Corresponding authors: Marwa Matboli[2], Medicinal Biochemistry Department, Ain Shams University School of Medicine. Marwasayed472@yahoo.com, DrMarwa_Matboly@med.asu.edu.eg

Supplementary tables

Supplementary **Table S3** Functional annotation table by Functional Annotation Tool DAVID Bioinformatics Resources 6.8, NIAID/NIH

Functional Annotation Table

2033	E1A binding protein p300(EP300)		Homo sapiens
BIOCARTA	CARM1 and Regulation of the Estrogen Receptor, Transcription Regulation by Methyltransferase of CARM1, Cell Cycle, Role of ERBB2 in Signal Transduction and Oncology, Hypoxia-Inducible Factor in the Cardiovascular System, IL-7 Signal Transduction, Role of MEF2D in T-cell Apoptosis, Melanocyte Development and Pigmentation Pathway, NFkB activation by Nontypeable Hemophilus influenzae, Hypoxia and p53 in the Cardiovascular system, Pelp1 Modulation of Estrogen Receptor Activity, Multi-step Regulation of Transcription by Pitx2, Mechanism of Gene Regulation by Peroxisome Proliferators via PPARa(alpha), Role of PPAR-gamma Coactivators in Obesity and Thermogenesis, Acetylation and Deacetylation of RelA in The Nucleus, TGF beta signaling pathway, Control of Gene Expression by Vitamin D Receptor,		
GOTERM_BP_DIRECT	negative regulation of transcription from RNA polymerase II promoter, response to hypoxia, somitogenesis, stimulatory C-type lectin receptor signaling pathway, transcription-coupled nucleotide-excision repair, regulation of transcription, DNA-templated, transcription from RNA polymerase II promoter, protein acetylation, internal protein amino acid acetylation, apoptotic process, DNA damage response, signal transduction by p53 class mediator resulting in cell cycle arrest, positive regulation of transcription from RNA polymerase II promoter involved in unfolded protein response, Notch signaling pathway, nervous system development, heart development, skeletal muscle tissue development, circadian rhythm, organ morphogenesis, regulation of autophagy, macrophage derived foam cell differentiation, viral process, N-terminal peptidyl-lysine acetylation, internal peptidyl-lysine acetylation, B cell differentiation, platelet formation, lung development, positive regulation of protein binding, positive regulation of type I interferon production, cellular response to UV, megakaryocyte development, intrinsic apoptotic signaling pathway in response to DNA damage by p53 class mediator, response to estrogen, positive regulation by host of viral transcription, histone H4 acetylation, histone H2B acetylation, fat cell differentiation, positive regulation of gene expression, epigenetic, positive regulation of transcription from RNA polymerase II promoter, protein stabilization, positive regulation of sequence-specific DNA binding transcription factor activity, regulation of cell cycle, regulation of androgen receptor signaling pathway, regulation of transcription from RNA polymerase II promoter in response to hypoxia, regulation of tubulin deacetylation, regulation of cellular		

	response to heat, regulation of signal transduction by p53 class mediator, beta-catenin-TCF complex assembly,
**GOTERM_CC_DIRECT**	histone acetyltransferase complex, nucleus, nucleoplasm, transcription factor complex, cytoplasm,
**GOTERM_MF_DIRECT**	RNA polymerase II core promoter sequence-specific DNA binding, core promoter binding, RNA polymerase II activating transcription factor binding, transcriptional activator activity, RNA polymerase II transcription regulatory region sequence-specific binding, p53 binding, DNA binding, chromatin binding, damaged DNA binding, transcription cofactor activity, transcription coactivator activity, histone acetyltransferase activity, lysine N-acetyltransferase activity, acting on acetyl phosphate as donor, protein binding, beta-catenin binding, protein C-terminus binding, transcription factor binding, zinc ion binding, acetyltransferase activity, transferase activity, transferring acyl groups, chromatin DNA binding, activating transcription factor binding, peptide N-acetyltransferase activity, nuclear hormone receptor binding, androgen receptor binding, pre-mRNA intronic binding,
**INTERPRO**	Zinc finger, TAZ-type, Zinc finger, ZZ-type, Bromodomain, Coactivator CBP, KIX domain, Nuclear receptor coactivator, interlocking, Domain of unknown function DUF902, CREBbp, Histone H3-K56 acetyltransferase, RTT109, Nuclear receptor coactivator, CREB-bp-like, interlocking, Bromodomain, conserved site,
**KEGG_PATHWAY**	cAMP signaling pathway, HIF-1 signaling pathway, FoxO signaling pathway, Cell cycle, Wnt signaling pathway, Notch signaling pathway, TGF-beta signaling pathway, Adherens junction, Jak-STAT signaling pathway, Long-term potentiation, Melanogenesis, Thyroid hormone signaling pathway, Glucagon signaling pathway, Huntington's disease, Tuberculosis, Hepatitis B, Influenza A, HTLV-I infection, Herpes simplex infection, Pathways in cancer, Viral carcinogenesis, MicroRNAs in cancer, Renal cell carcinoma, Prostate cancer,
**OMIM_DISEASE**	Colorectal cancer, somatic, Rubinstein-Taybi syndrome 2,
**SMART**	ZnF_ZZ, BROMO, ZnF_TAZ, SM01250,
**UP_KEYWORDS**	3D-structure, Acetylation, Acyltransferase, Biological rhythms, Bromodomain, Cell cycle, Chromosomal rearrangement, Citrullination, Coiled coil, Complete proteome, Cytoplasm, Direct protein sequencing, Disease mutation, Host-virus interaction, Isopeptide bond, Metal-binding, Methylation, Nucleus, Phosphoprotein, Polymorphism, Proteomics identification, Reference

	proteome, Repeat, Transcription, Transcription regulation, Transferase, Ubl conjugation, Zinc, Zinc-finger,		
**UP_SEQ_FEATURE**	chain:Histone acetyltransferase p300, compositionally biased region:Poly-Gln, compositionally biased region:Poly-Glu, compositionally biased region:Poly-Ser, domain:Bromo, domain:KIX, helix, modified residue, mutagenesis site, region of interest:Binding region for E1A adenovirus, region of interest:Interaction with HTLV-1 Tax, region of interest:Interaction with NCOA2, sequence conflict, sequence variant, short sequence motif:Nuclear localization signal, site:Breakpoint for translocation to form MYST3-EP300 and EP300-MYST3, site:Interaction with NCOA2, strand, turn, zinc finger region:TAZ-type 1, zinc finger region:TAZ-type 2, zinc finger region:ZZ-type,		
**1906**	**endothelin 1(EDN1)**		**Homo sapiens**
**BIOCARTA**	Role of EGF Receptor Transactivation by GPCRs in Cardiac Hypertrophy, Hypoxia-Inducible Factor in the Cardiovascular System, NFAT and Hypertrophy of the heart (Transcription in the broken heart),		
**GOTERM_BP_DIRECT**	negative regulation of transcription from RNA polymerase II promoter, prostaglandin biosynthetic process, patterning of blood vessels, in utero embryonic development, histamine secretion, regulation of systemic arterial blood pressure by endothelin, regulation of pH, cell surface receptor signaling pathway, G-protein coupled receptor signaling pathway, positive regulation of cytosolic calcium ion concentration, protein kinase C-activating G-protein coupled receptor signaling pathway, cell-cell signaling, heart development, respiratory gaseous exchange, body fluid secretion, regulation of blood pressure, positive regulation of cell proliferation, dorsal/ventral pattern formation, response to ozone, multicellular organism aging, positive regulation of heart rate, positive regulation of endothelial cell migration, positive regulation of cardiac muscle hypertrophy, positive regulation of receptor biosynthetic process, neural crest cell development, phosphatidylinositol 3-kinase signaling, response to activity, artery smooth muscle contraction, vein smooth muscle contraction, glucose transport, cell growth, regulation of vasoconstriction, sensory perception of pain, calcium-mediated signaling, peptide hormone secretion, nitric oxide transport, negative regulation of blood coagulation, positive regulation of cell migration, neutrophil chemotaxis, negative regulation of cAMP biosynthetic process, phospholipase D-activating G-protein coupled receptor signaling pathway, negative regulation of cellular protein metabolic process, positive regulation of prostaglandin secretion, response to lipopolysaccharide, response to testosterone, negative regulation of smooth muscle cell apoptotic process, response to prostaglandin F, response to nicotine, intracellular signal transduction, cellular response to drug, positive regulation of urine volume, positive regulation of renal sodium excretion, response to muscle stretch, epithelial fluid transport, vasoconstriction, protein kinase C deactivation, middle ear morphogenesis, positive regulation of odontogenesis, superoxide anion generation, rhythmic excitation, response to amino acid, positive		

	regulation of MAP kinase activity, positive regulation of JUN kinase activity, response to leptin, leukocyte activation, positive regulation of nitric oxide biosynthetic process, positive regulation of cell size, positive regulation of mitotic nuclear division, positive regulation of transcription from RNA polymerase II promoter, positive regulation of smooth muscle contraction, positive regulation of hormone secretion, negative regulation of hormone secretion, inositol phosphate-mediated signaling, positive regulation of smooth muscle cell proliferation, positive regulation of sequence-specific DNA binding transcription factor activity, cartilage development, positive regulation of cytosolic calcium ion concentration involved in phospholipase C-activating G-protein coupled signaling pathway, negative regulation of nitric-oxide synthase biosynthetic process, membrane depolarization, regulation of sensory perception of pain, maternal process involved in parturition, positive regulation of sarcomere organization, positive regulation of prostaglandin-endoperoxide synthase activity, positive regulation of cell growth involved in cardiac muscle cell development, positive regulation of chemokine-mediated signaling pathway, cellular response to calcium ion, cellular response to interferon-gamma, cellular response to interleukin-1, cellular response to tumor necrosis factor, cellular response to peptide hormone stimulus, cellular response to glucocorticoid stimulus, cellular response to mineralocorticoid stimulus, cellular response to fatty acid, cellular response to hypoxia, response to dexamethasone, cellular response to transforming growth factor beta stimulus, response to salt,
**GOTERM_CC_DIRECT**	extracellular region, extracellular space, cytoplasm, Weibel-Palade body, basal part of cell, rough endoplasmic reticulum lumen,
**GOTERM_MF_DIRECT**	cytokine activity, hormone activity, protein binding, endothelin A receptor binding, endothelin B receptor binding,
**INTERPRO**	Endothelin-like toxin, Endothelin-like toxin, conserved site, Bibrotoxin/Sarafotoxin-D,
**KEGG_PATHWAY**	HIF-1 signaling pathway, TNF signaling pathway, Melanogenesis,
**OMIM_DISEASE**	Question mark ears, isolated, Auriculocondylar syndrome 3, High density lipoprotein cholesterol level QTL 7,

**SMART**	END,		
**UP_KEYWORDS**	3D-structure, Cleavage on pair of basic residues, Complete proteome, Direct protein sequencing, Disease mutation, Disulfide bond, Polymorphism, Reference proteome, Secreted, Signal, Vasoactive, Vasoconstrictor,		
**UP_SEQ_FEATURE**	disulfide bond, helix, peptide:Big endothelin-1, peptide:Endothelin-1, region of interest:Endothelin-like, sequence variant, signal peptide, strand,		
**7124**	**tumor necrosis factor(TNF)**		**Homo sapiens**
**BBID**	110.CRHandLC-NA, 111.Stress_influences_immunity, 112.StressandCRHinfluence, 114.Genomic_reformatting_Brain_Ischemia, 13.MS_progression, 18.Cytokine_astocytes, 19.Cytokine_microglia, 38.Cell_cycle_arrest_and_apoptosis_ceramide, 40.Deg_of_Chrom_DNA_TNF-ind_apoptosis, 56.Macrophage_regulation_of_CD4+T_cells, 58.(CD40L)_immnosurveillance, 60.IL-15_rheumatoid-arthritis_synovitis, 77.IkBa_Kinase_JNK_MEKK1, 8.MonocytesIFNF_IL4, 88.Alternatively_Activated_APC, 97.Immune_injury_MS-lesions_MS_antigen,		
**BIOCARTA**	Cadmium induces DNA synthesis and proliferation in macrophages, Cytokine Network, Free Radical Induced Apoptosis, Adhesion and Diapedesis of Granulocytes, HIV-I Nef, Stress Induction of HSP Regulation, IL-10 Anti-inflammatory Signaling Pathway, Signal transduction through IL1R, Cytokines and Inflammatory Response, Keratinocyte Differentiation, Cells and Molecules involved in local acute inflammatory response, Msp/Ron Receptor Signaling Pathway, NF-kB Signaling Pathway, NFkB activation by Nontypeable Hemophilus influenzae, Regulation of transcriptional activity by PML, Mechanism of Gene Regulation by Peroxisome Proliferators via PPARa(alpha), Acetylation and Deacetylation of RelA in The Nucleus, SODD/TNFR1 Signaling Pathway, TNF/Stress Related Signaling, Chaperones modulate interferon Signaling Pathway, TNFR1 Signaling Pathway, Visceral Fat Deposits and the Metabolic Syndrome,		
**GOTERM_BP_DIRECT**	protein import into nucleus, translocation, negative regulation of transcription from RNA polymerase II promoter, MAPK cascade, activation of MAPKKK activity, activation of MAPK activity, positive regulation of cytokine production, positive regulation of protein phosphorylation, chronic inflammatory response to antigenic stimulus, negative regulation of cytokine secretion involved in immune response, positive regulation of chronic inflammatory response to antigenic stimulus, positive regulation of humoral immune response mediated by circulating		

immunoglobulin, glucose metabolic process, activation of cysteine-type endopeptidase activity involved in apoptotic process, inflammatory response, immune response, humoral immune response, I-kappaB kinase/NF-kappaB signaling, JNK cascade, extrinsic apoptotic signaling pathway via death domain receptors, intrinsic apoptotic signaling pathway in response to DNA damage, response to virus, response to salt stress, positive regulation of gene expression, negative regulation of gene expression, negative regulation of alkaline phosphatase activity, regulation of tumor necrosis factor-mediated signaling pathway, negative regulation of lipid storage, extracellular matrix organization, osteoclast differentiation, sequestering of triglyceride, cortical actin cytoskeleton organization, positive regulation of protein complex assembly, positive regulation of fever generation, lipopolysaccharide-mediated signaling pathway, negative regulation of interleukin-6 production, positive regulation of chemokine production, positive regulation of interferon-gamma production, positive regulation of interleukin-6 production, positive regulation of interleukin-8 production, receptor biosynthetic process, positive regulation of peptidyl-serine phosphorylation, tumor necrosis factor-mediated signaling pathway, positive regulation of heterotypic cell-cell adhesion, negative regulation of myosin-light-chain-phosphatase activity, regulation of cell proliferation, positive regulation of NF-kappaB import into nucleus, positive regulation of apoptotic process, positive regulation of programmed cell death, regulation of I-kappaB kinase/NF-kappaB signaling, positive regulation of I-kappaB kinase/NF-kappaB signaling, negative regulation of protein complex disassembly, positive regulation of protein complex disassembly, positive regulation of cysteine-type endopeptidase activity involved in apoptotic process, positive regulation of MAP kinase activity, protein kinase B signaling, positive regulation of JUN kinase activity, negative regulation of growth of symbiont in host, negative regulation of viral genome replication, positive regulation of chemokine biosynthetic process, cellular extravasation, positive regulation of interleukin-8 biosynthetic process, positive regulation of nitric oxide biosynthetic process, negative regulation of fat cell differentiation, negative regulation of myoblast differentiation, negative regulation of osteoblast differentiation, regulation of osteoclast differentiation, positive regulation of osteoclast differentiation, positive regulation of cell adhesion, positive regulation of protein kinase activity, negative regulation of transcription, DNA-templated, positive regulation of transcription, DNA-templated, positive regulation of transcription from RNA polymerase II promoter, positive regulation of translational initiation by iron, negative regulation of glucose import, positive regulation of JNK cascade, embryonic digestive tract development, positive regulation of smooth muscle cell proliferation, positive regulation of cytokine secretion, positive regulation of phagocytosis, regulation of insulin secretion, defense response to Gram-positive bacterium, leukocyte tethering or rolling, negative regulation of lipid catabolic process, regulation of immunoglobulin secretion, positive regulation of membrane protein ectodomain proteolysis, positive regulation of sequence-specific DNA binding transcription factor activity, positive regulation of NF-kappaB transcription factor activity, positive regulation of protein transport, response to glucocorticoid, positive regulation of NFAT protein import into nucleus, positive regulation of hair follicle development, positive regulation of protein kinase B signaling, positive regulation of vitamin D biosynthetic process, positive regulation of calcidiol 1-

	monooxygenase activity, epithelial cell proliferation involved in salivary gland morphogenesis, regulation of branching involved in salivary gland morphogenesis, negative regulation of branching involved in lung morphogenesis, positive regulation of ERK1 and ERK2 cascade, cellular response to lipopolysaccharide, cellular response to amino acid stimulus, cellular response to nicotine, cellular response to organic cyclic compound, death-inducing signaling complex assembly, positive regulation of mononuclear cell migration, positive regulation of podosome assembly, protein localization to plasma membrane, establishment of protein localization to plasma membrane, extrinsic apoptotic signaling pathway, necroptotic signaling pathway, positive regulation of NIK/NF-kappaB signaling, positive regulation of superoxide dismutase activity, regulation of establishment of endothelial barrier, negative regulation of bicellular tight junction assembly, positive regulation of leukocyte adhesion to vascular endothelial cell, positive regulation of leukocyte adhesion to arterial endothelial cell, positive regulation of protein localization to cell surface, positive regulation of ceramide biosynthetic process, positive regulation of blood microparticle formation, positive regulation of chemokine (C-X-C motif) ligand 2 production, regulation of reactive oxygen species metabolic process, negative regulation of extrinsic apoptotic signaling pathway in absence of ligand,
**GOTERM_CC_DIRECT**	phagocytic cup, extracellular region, extracellular space, plasma membrane, integral component of plasma membrane, external side of plasma membrane, cell surface, membrane, integral component of membrane, membrane raft, recycling endosome,
**GOTERM_MF_DIRECT**	protease binding, cytokine activity, tumor necrosis factor receptor binding, protein binding, identical protein binding, transcription regulatory region DNA binding,
**INTERPRO**	Tumour necrosis factor alpha/cachectin, Tumour necrosis factor, Tumour necrosis factor alpha/beta/c, Tumour necrosis factor-like domain, Tumour necrosis factor, conserved site,
**KEGG_PATHWAY**	MAPK signaling pathway, Cytokine-cytokine receptor interaction, NF-kappa B signaling pathway, Sphingolipid signaling pathway, mTOR signaling pathway, Apoptosis, TGF-beta signaling pathway, Osteoclast differentiation, Antigen processing and presentation, Toll-like receptor signaling pathway, NOD-like receptor signaling pathway, RIG-I-like receptor signaling pathway, Hematopoietic cell lineage, Natural killer cell mediated cytotoxicity, T cell receptor signaling pathway, Fc epsilon RI signaling pathway, TNF signaling pathway, Adipocytokine signaling pathway, Type II diabetes mellitus, Insulin resistance, Non-alcoholic fatty liver disease (NAFLD), Type I diabetes mellitus, Alzheimer's disease, Amyotrophic lateral sclerosis (ALS), Pertussis, Legionellosis, Leishmaniasis, Chagas disease (American trypanosomiasis), African

	trypanosomiasis, Malaria, Toxoplasmosis, Amoebiasis, Tuberculosis, Hepatitis C, Hepatitis B, Influenza A, HTLV-I infection, Herpes simplex infection, Proteoglycans in cancer, Asthma, Inflammatory bowel disease (IBD), Systemic lupus erythematosus, Rheumatoid arthritis, Allograft rejection, Graft-versus-host disease, Hypertrophic cardiomyopathy (HCM), Dilated cardiomyopathy,
**OMIM_DISEASE**	Migraine without aura, susceptibility to, Asthma, susceptibility to, Malaria, cerebral, susceptibility to, Dementia, vascular, susceptibility to, Septic shock, susceptibility to,
**SMART**	TNF,
**UP_KEYWORDS**	3D-structure, Cell membrane, Complete proteome, Cytokine, Direct protein sequencing, Disulfide bond, Glycoprotein, Lipoprotein, Membrane, Myristate, Phosphoprotein, Polymorphism, Proteomics identification, Reference proteome, Secreted, Signal-anchor, Transmembrane, Transmembrane helix,
**UP_SEQ_FEATURE**	chain:Tumor necrosis factor, membrane form, chain:Tumor necrosis factor, soluble form, disulfide bond, glycosylation site:O-linked (GalNAc...); in soluble form, helix, lipid moiety-binding region:N6-myristoyl lysine, modified residue, mutagenesis site, sequence conflict, sequence variant, site:Cleavage; by ADAM17, strand, topological domain:Cytoplasmic, topological domain:Extracellular, transmembrane region,

Table S4 Putative interaction between miRNA-6888-5p and the selected mRNA by Target scan database

miRNA	Position in the UTR	seed match	context++ score	context++ score percentile	weighted context++ score	conserved branch length	Pct
EDN1							
hsa-miR-6888	586-592	7mer-m8	-0.24	93	-0.24	3.574	0.55
hsa-miR-6888	1022-1029	8mer	-0.37	98	-0.37	2.185	N/A
EP300							
hsa-miR-6888	1113-1119	7mer-m8	-0.1	77	-0.01	0.223	N/A
MAPK3							
hsa-miR-6888	173-179	7mer-m8	-0.09	75	-0.09	0	N/A
TNF							
hsa-miR-6888	173-179	7mer-m8	-0.09	75	-0.09	0	N/A

Table S5 Details of the GSE89632 datasets retrieved from the GEO database

GEO dataset	Platform	No. of cases	Organism
GSE89632	GPL14951<br>Illumina HumanHT-12 WG-DASL V4.0 R2 expression beadchip	HC, 24<br>NASH, 19	Homo sapiens

Abbreviations: GEO, Gene Expression Omnibus; HC, healthy control; SS, steatosis; NASH, non-alcoholic steatohepatitis

Table S6 Reagents used in the biochemical parameters assessment:

Biochemical Test	Reagent
serum ALT	OSR6607
Serum AST	OSR6509
Serum GGT	OSR6119
total bilirubin	OSR6112
direct bilirubin	OSR6111
Serum albumin	OSR6102
LDL Cholesterol	OSR6196
HDL cholesterol	OSR6195
total cholesterol	OSR6116
triglycerides	OSR60118
fasting blood glucose	OSR6121
HbA1C	OSR6192

Table S7 List of primers used for RT-PCR:

Primer Assay	GeneGlobe ID
TNF (NM_000594)	QT00029162
MAPK3 (NM_001109891)	QT02589314
EP300 (NM_001429)	QT00094500
EDN1 (NM_001955)	QT00088235
GAPDH (NM_001256799)	QT00079247
Hs_miR-6888-5p_1	MS00048069
Hs_SNORD72_11	MS00033719
RABGAP1L-IT1 (ENST00000414890)	SBH0671985
HS_GAD1_1390172	SBH0146388

Supplementary figures:

Figures S1: Verification of the differential expression of the selected genes from other GEO datasets
Figure S1: A; Scatter plot for the selected genes from GSE60349 (I; EDN1, II;EP300, III;MAPK3, IV;TNF), B; Scatter plot for the selected genes from GSE35961(I; EDN1, II;EP300, III;MAPK3, IV;TNF),

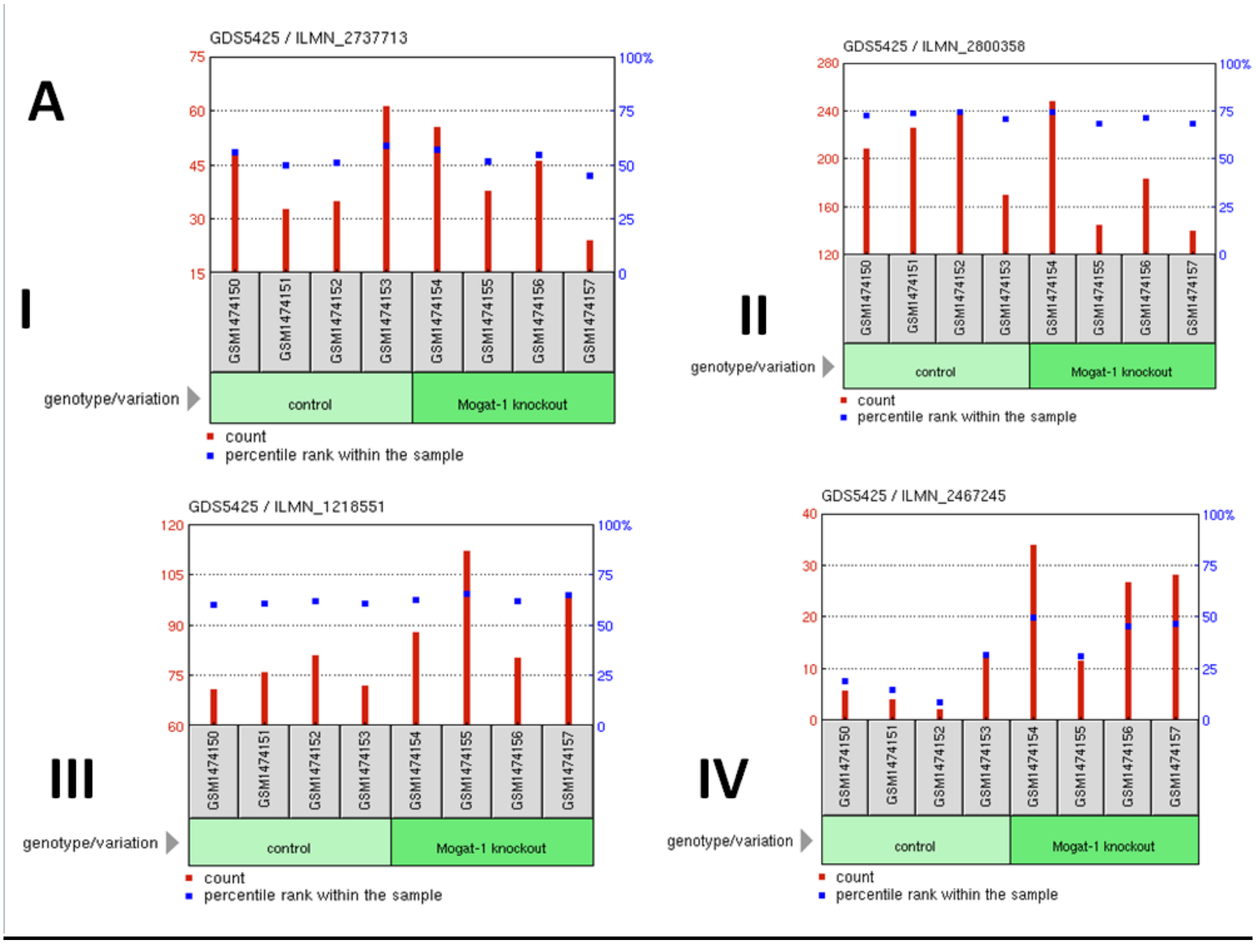
A
I
GDS5425 / ILMN_2737713
II
GDS5425 / ILMN_2800358
III
GDS5425 / ILMN_1218551
IV
GDS5425 / ILMN_2467245
GSM1474150
GSM1474151
GSM1474152
GSM1474153
GSM1474154
GSM1474155
GSM1474156
GSM1474157
genotype/variation
control
Mogat-1 knockout
count
percentile rank within the sample

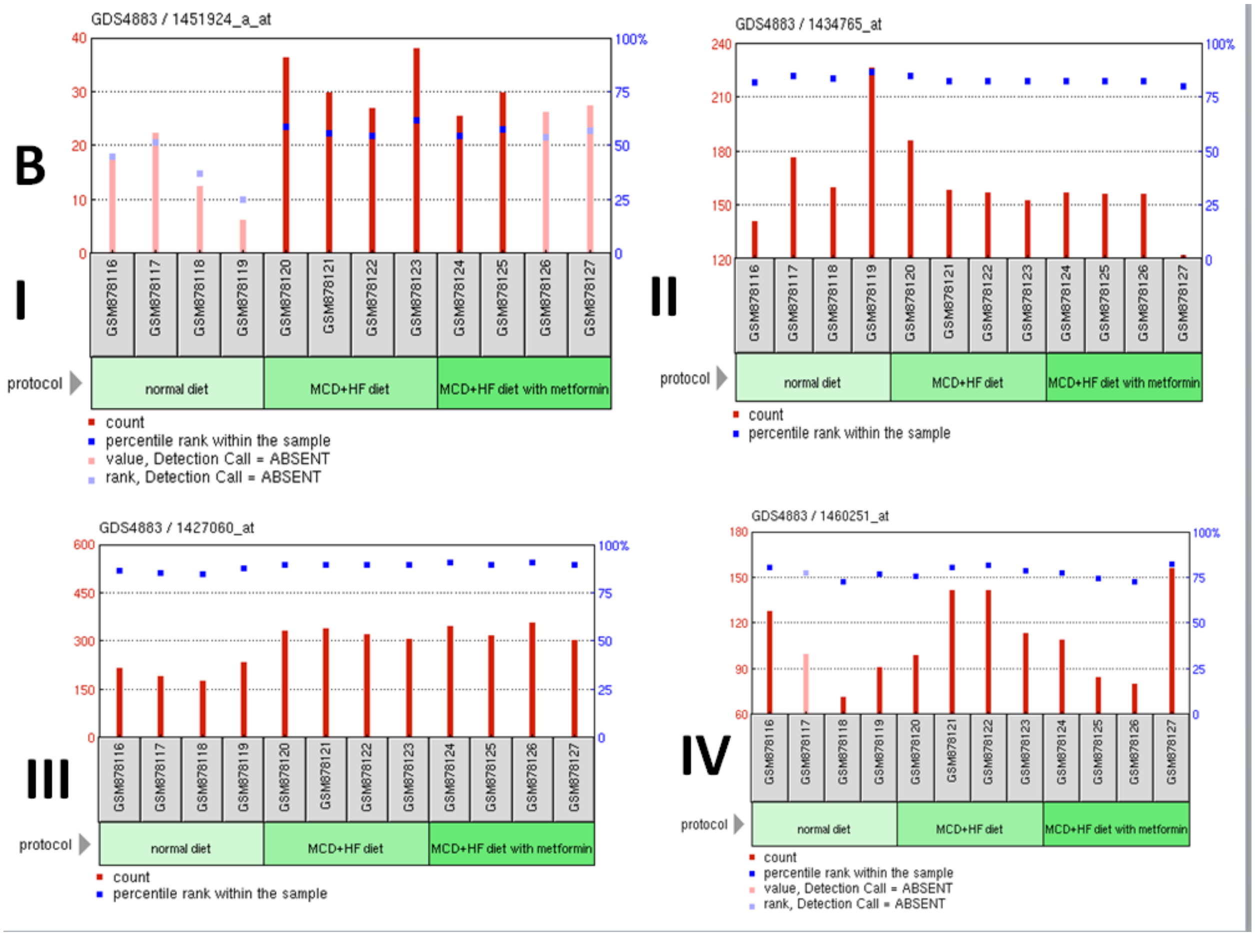
B
I
GDS4883 / 1451924_a_at
II
GDS4883 / 1434765_at
III
GDS4883 / 1427060_at
IV
GDS4883 / 1460251_at
GSM878116
GSM878117
GSM878118
GSM878119
GSM878120
GSM878121
GSM878122
GSM878123
GSM878124
GSM878125
GSM878126
GSM878127
protocol
normal diet
MCD+HF diet
MCD+HF diet with metformin
count
percentile rank within the sample
value, Detection Call = ABSENT
rank, Detection Call = ABSENT

Figures S2: Validation of the relation between EDN1, *EP300* ,*MAPK3, &* TNF, genes to NAFLD/NASH pathogenesis, B cell proliferation/Cytokine response by public microarray databases

I. Validation of EDN1, *EP300* ,*MAPK3, &* TNF, genes to NAFLD/NASH pathogenesis in different GEO datasets

II. Gene ontology of EDN1, EP300 ,MAPK3, & TNF, mRNA by KEGG map that was retrieved from GeneCards®: The Human Gene Database .. It shows that the chosen mRNAs are linked specifically to Cytokine response

III. Gene expression of EDN1, EP300 ,MAPK3, & TNF, mRNA in liver that was retrieved from Gene – NCBI database. X axis represents level of RNA expression while Y axis represents the tissue in which the gene is expressed.

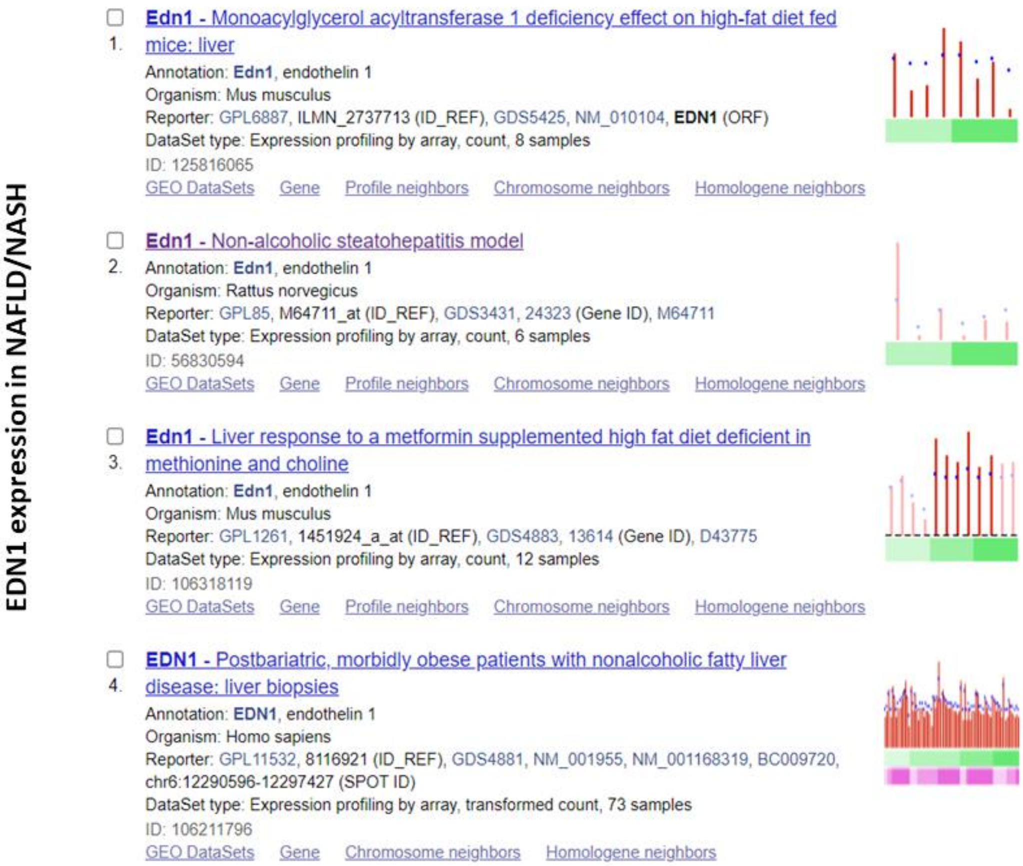

EDN1 Gene ontology

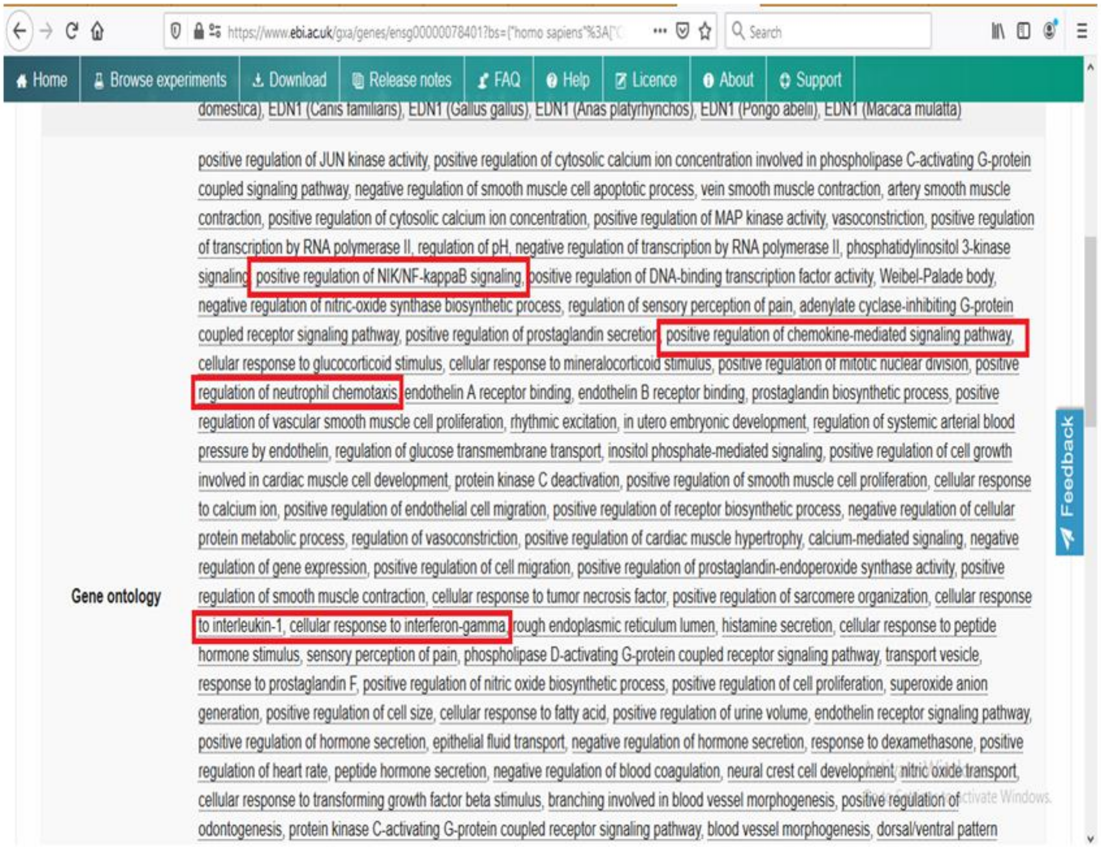

EDN1 expression in different tissues especially liver

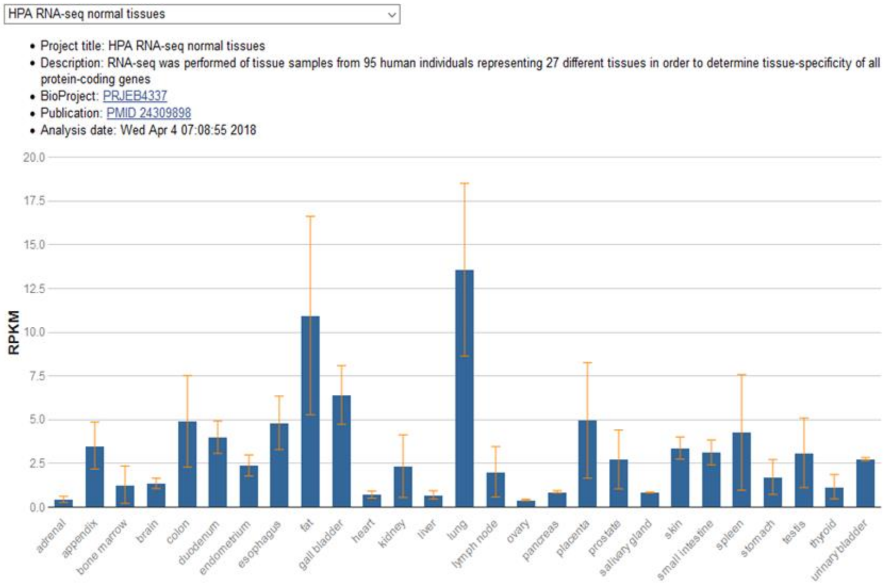

Ep300 expression in NAFLD/NASH

☐ 1. **Ep300** - Monoacylglycerol acyltransferase 1 deficiency effect on high-fat diet fed mice: liver

Annotation: **Ep300**, E1A binding protein p300
Organism: Mus musculus
Reporter: GPL6887, ILMN_2800358 (ID_REF), GDS5425, NM_177821, **EP300** (ORF)
DataSet type: Expression profiling by array, count, 8 samples
ID: 125786997
GEO DataSets Gene Profile neighbors Chromosome neighbors Homologene neighbors

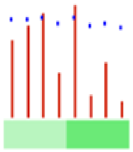

☐ 2. Eid1 - Non-alcoholic steatohepatitis model

Annotation: Eid1, **EP300** interacting inhibitor of differentiation 1
Organism: Rattus norvegicus
Reporter: GPL85, rc_AA892137_at (ID_REF), GDS3431, 499882 (Gene ID), AA892137
DataSet type: Expression profiling by array, count, 6 samples
ID: 56832211
GEO DataSets Gene Profile neighbors Chromosome neighbors Homologene neighbors

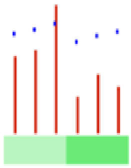

☐ 3. **Ep300** - Monoacylglycerol acyltransferase 1 deficiency effect on high-fat diet fed mice: liver

Annotation: **Ep300**, E1A binding protein p300
Organism: Mus musculus
Reporter: GPL6887, ILMN_2561221 (ID_REF), GDS5425, AK040384, A430090G16RIK (ORF)
DataSet type: Expression profiling by array, count, 8 samples
ID: 125815211
GEO DataSets Gene Profile neighbors Chromosome neighbors Homologene neighbors

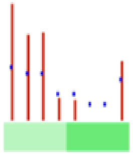

☐ 4. Eid2 - Monoacylglycerol acyltransferase 1 deficiency effect on high-fat diet fed mice: liver

Annotation: Eid2, **EP300** interacting inhibitor of differentiation 2
Organism: Mus musculus
Reporter: GPL6887, ILMN_1223895 (ID_REF), GDS5425, NM_198425, EID2 (ORF)
DataSet type: Expression profiling by array, count, 8 samples
ID: 125776135
GEO DataSets Gene Profile neighbors Chromosome neighbors Homologene neighbors

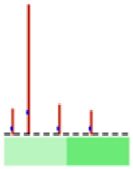

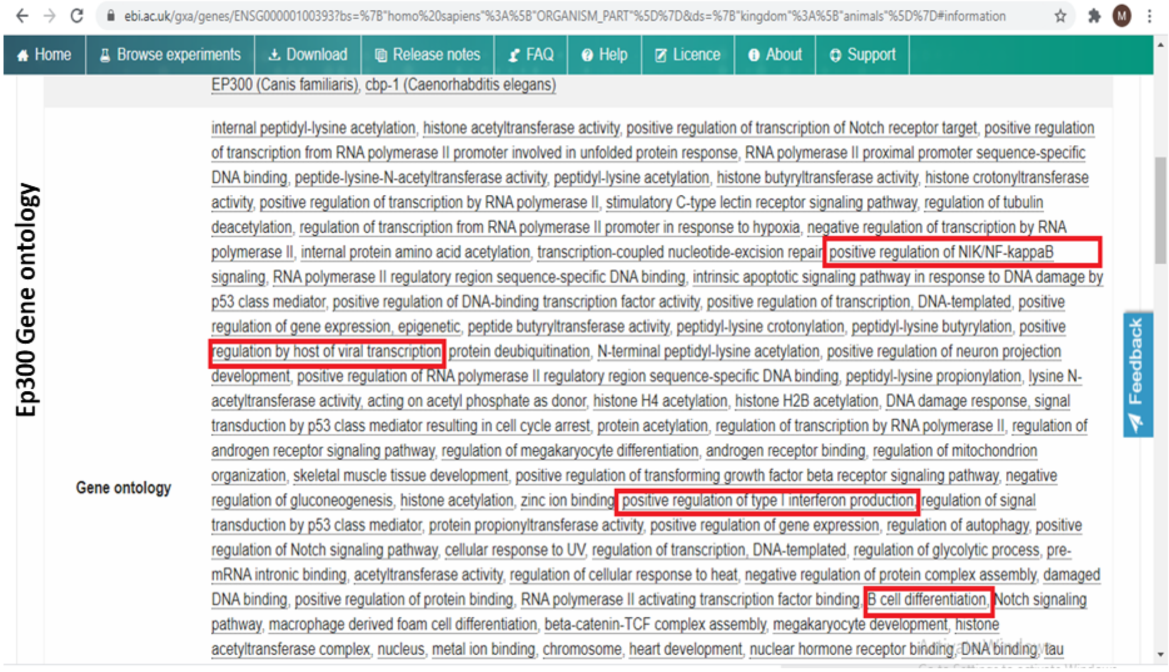

Ep300 expression in different tissues especially liver

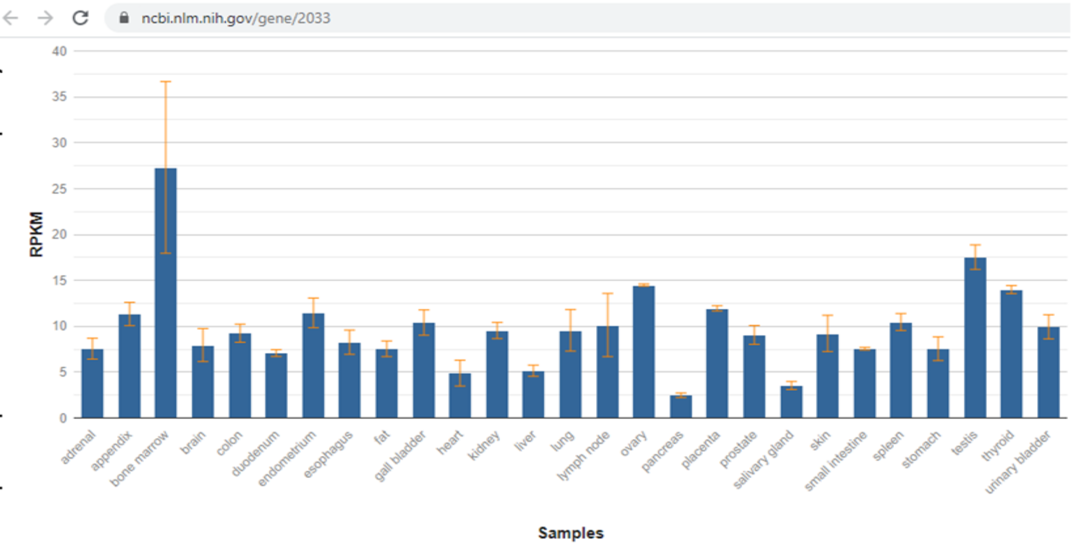

MAPK3 expression in NAFLD/NASH

1. Mapk3 - Non-alcoholic steatohepatitis model
Annotation: **Mapk3**, mitogen activated protein kinase 3
Organism: Rattus norvegicus
Reporter: GPL85, M61177_s_at (ID_REF), GDS3431, 50689 (Gene ID), M61177
DataSet type: Expression profiling by array, count, 6 samples
ID: 56830541
GEO DataSets Gene Profile neighbors Chromosome neighbors Homologene neighbors

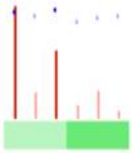

2. Mapk3 - Monoacylglycerol acyltransferase 1 deficiency effect on high-fat diet fed mice: liver
Annotation: **Mapk3**, mitogen-activated protein kinase 3
Organism: Mus musculus
Reporter: GPL6887, ILMN_1218551 (ID_REF), GDS5425, NM_011952, **MAPK3** (ORF)
DataSet type: Expression profiling by array, count, 8 samples
ID: 125772794
GEO DataSets Gene Profile neighbors Chromosome neighbors Homologene neighbors

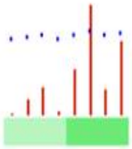

3. Mapk3 - Monoacylglycerol acyltransferase 1 deficiency effect on high-fat diet fed mice: liver
Annotation: **Mapk3**, mitogen-activated protein kinase 3
Organism: Mus musculus
Reporter: GPL6887, ILMN_2715744 (ID_REF), GDS5425, NM_011952, **MAPK3** (ORF)
DataSet type: Expression profiling by array, count, 8 samples
ID: 125810648
GEO DataSets Gene Profile neighbors Chromosome neighbors Homologene neighbors

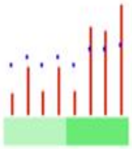

4. Mapk3 - Liver response to a metformin supplemented high fat diet deficient in methionine and choline
Annotation: **Mapk3**, mitogen-activated protein kinase 3
Organism: Mus musculus
Reporter: GPL1261, 1427060_at (ID_REF), GDS4883, 26417 (Gene ID), BI414398
DataSet type: Expression profiling by array, count, 12 samples
ID: 106293266
GEO DataSets Gene Profile neighbors Chromosome neighbors Homologene neighbors

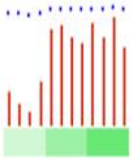

5. MAPK3 - Postbariatric, morbidly obese patients with nonalcoholic fatty liver disease: liver biopsies
Annotation: **MAPK3**, mitogen-activated protein kinase 3
Organism: Homo sapiens
Reporter: GPL11532, 8000811 (ID_REF), GDS4881, NM_001040056, NM_002746,

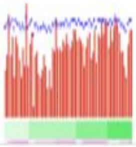

MAPK3 Gene ontology

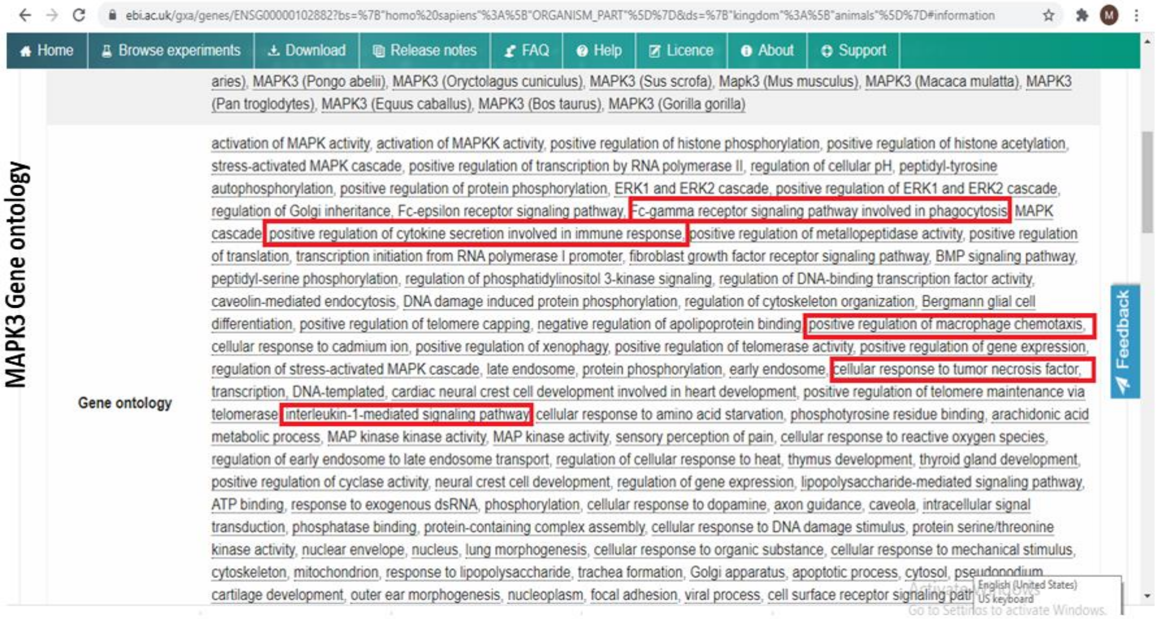

MAPK3 expression in different tissues especially liver

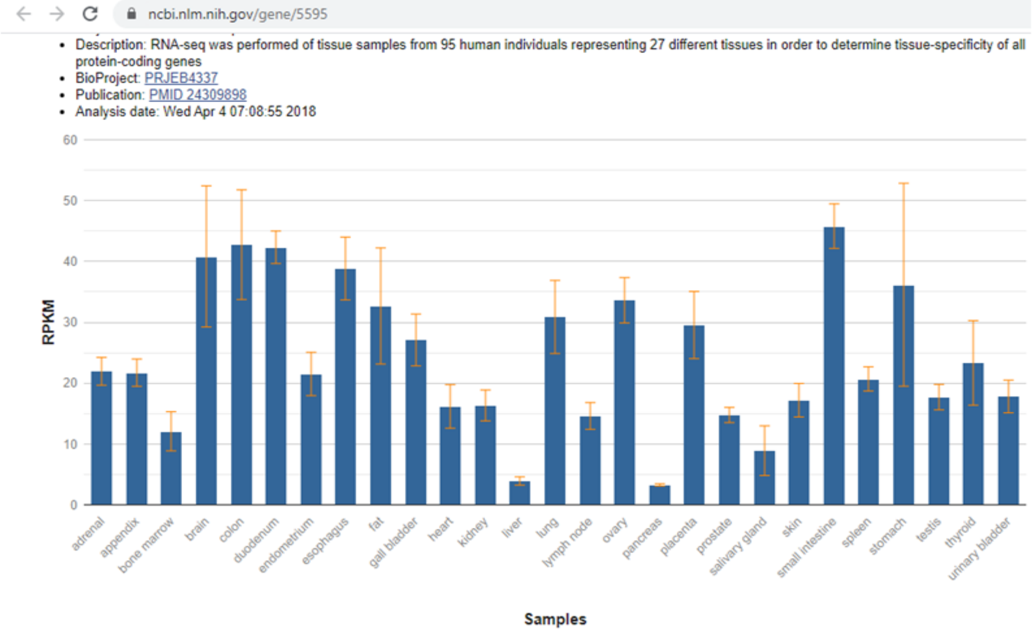

TNFα expression in NAFLD/NASH

1. [Tnf - Monoacylglycerol acyltransferase 1 deficiency effect on high-fat diet fed mice: liver]
Annotation: Tnf, tumor necrosis factor
Organism: Mus musculus
Reporter: GPL6887, ILMN_2467245 (ID_REF), GDS5425, NM_013693, TNF (ORF)
DataSet type: Expression profiling by array, count, 8 samples
ID: 125816732
GEO DataSets Gene Profile neighbors Chromosome neighbors Homologene neighbors

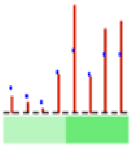

2. [Tnf - Monoacylglycerol acyltransferase 1 deficiency effect on high-fat diet fed mice: liver]
Annotation: Tnf, tumor necrosis factor
Organism: Mus musculus
Reporter: GPL6887, ILMN_2899863 (ID_REF), GDS5425, NM_013693, TNF (ORF)
DataSet type: Expression profiling by array, count, 8 samples
ID: 125785899
GEO DataSets Gene Profile neighbors Chromosome neighbors Homologene neighbors

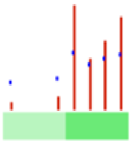

3. [Tnf - Non-alcoholic steatohepatitis model]
Annotation: Tnf, tumor necrosis factor
Organism: Rattus norvegicus
Reporter: GPL85, L00981mRNA#2_at (ID_REF), GDS3431, 24835 (Gene ID), L00981
DataSet type: Expression profiling by array, count, 6 samples
ID: 56829638
GEO DataSets Gene Profile neighbors Chromosome neighbors Homologene neighbors

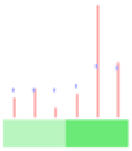

4. [Tnf - Non-alcoholic steatohepatitis model]
Annotation: Tnf, tumor necrosis factor
Organism: Rattus norvegicus
Reporter: GPL85, E02468cds_s_at (ID_REF), GDS3431, 24835 (Gene ID), E02468
DataSet type: Expression profiling by array, count, 6 samples
ID: 56829379
GEO DataSets Gene Profile neighbors Chromosome neighbors Homologene neighbors

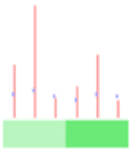

5. [Tnf - Liver response to a metformin supplemented high fat diet deficient in methionine and choline]
Annotation: Tnf, tumor necrosis factor
Organism: Mus musculus
Reporter: GPL1261, 1419607_at (ID_REF), GDS4883, 21926 (Gene ID), NM_013693
DataSet type: Expression profiling by array, count, 12 samples

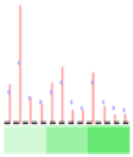

TNFα Gene ontology

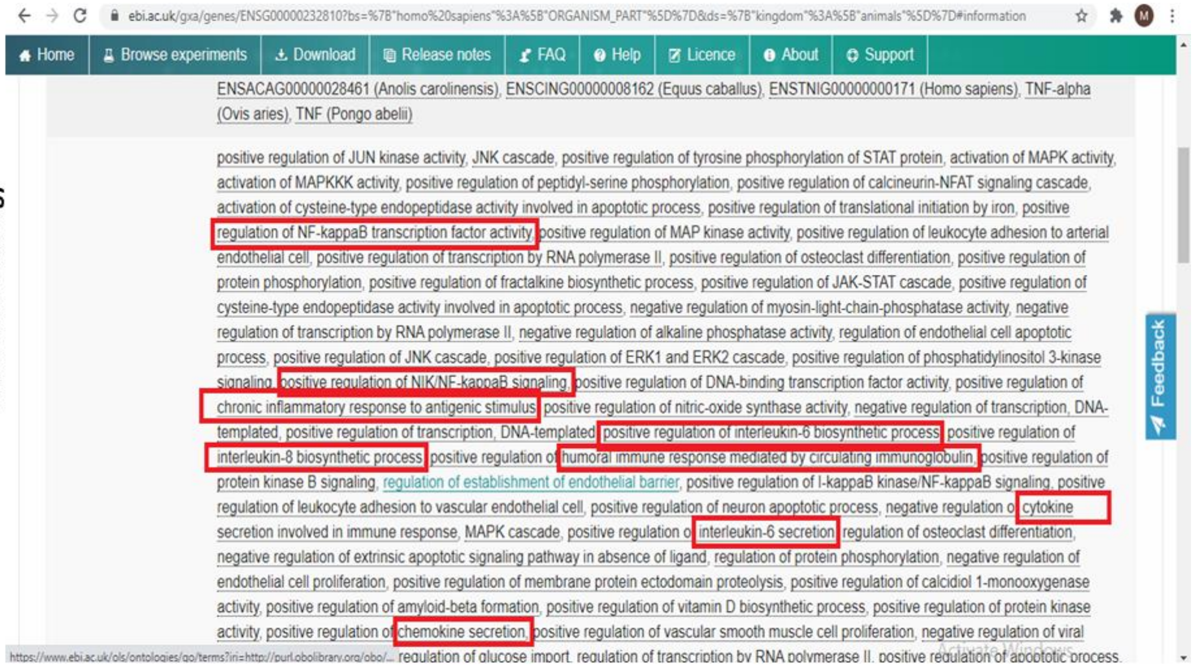

TNFα expression in different tissues especially liver

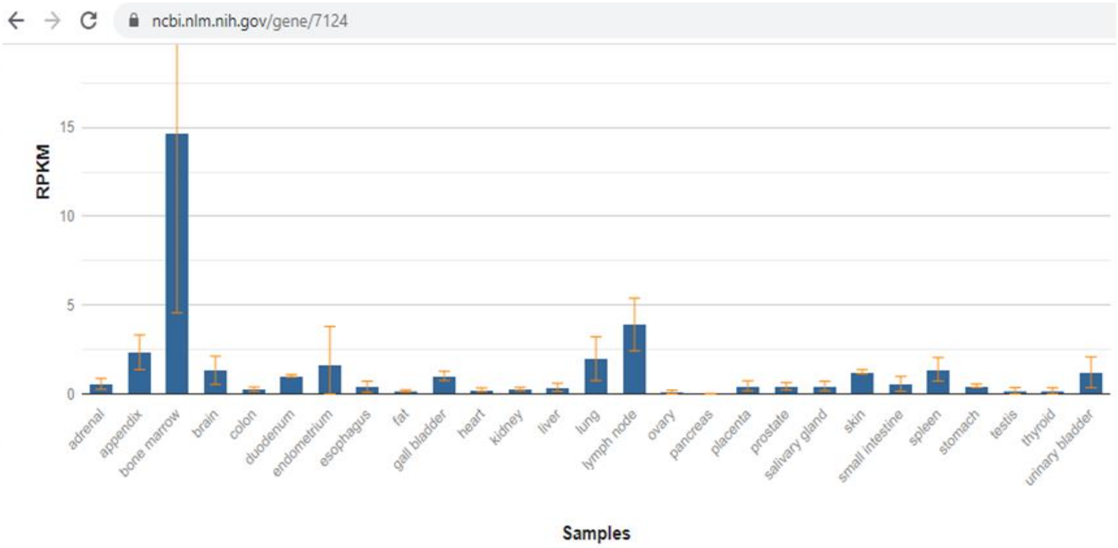

Figures S3: Validation of the association of EDN1, *EP300* ,*MAPK3*, *&* TNF with STING-mediated cytokine signaling in KEGG map , and STING-database .

The protein–protein interaction networks that was obtained by using String tool. DEGs

Validation of the association of EDN1, *EP300* ,*MAPK3*, *&* TNF with STING-mediated cytokine signaling in KEGG map

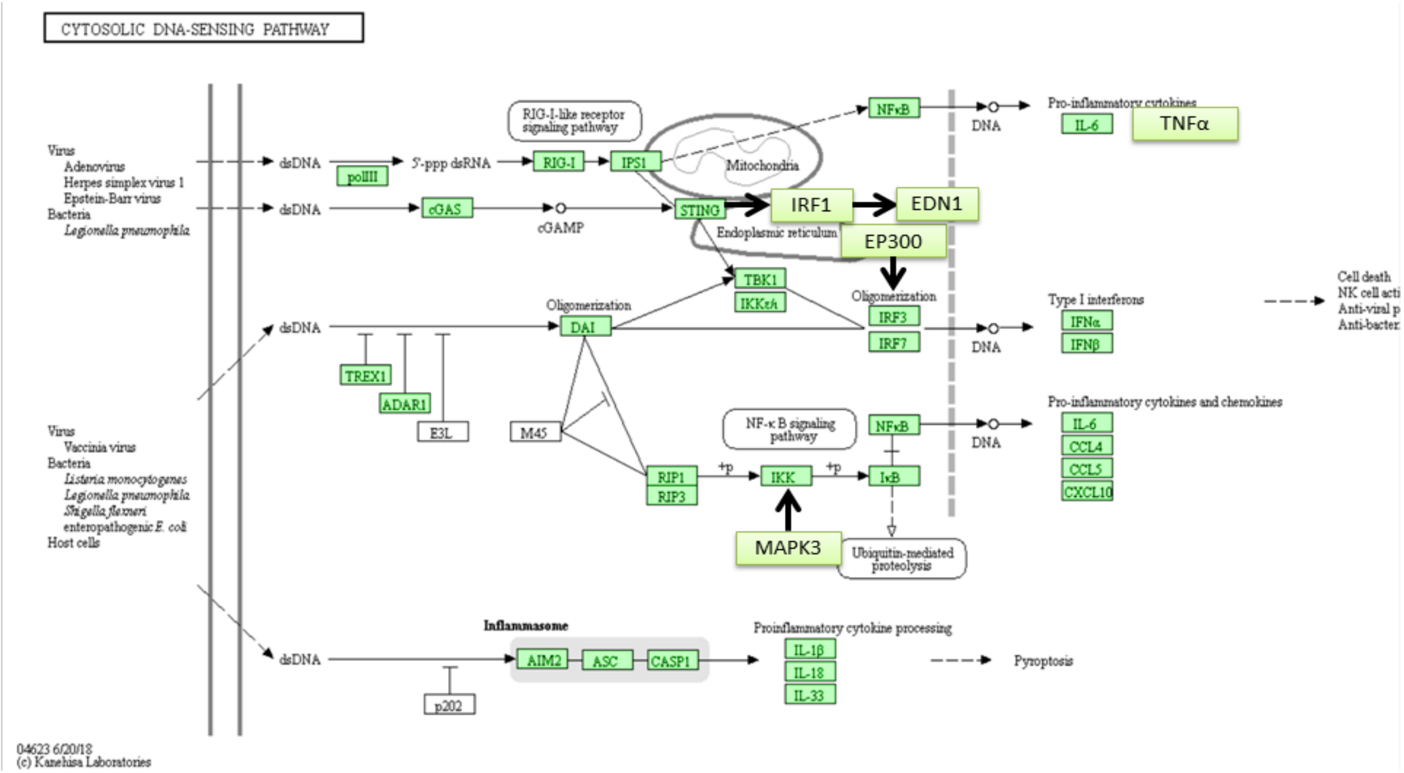

Validation of the association of EDN1, *EP300* ,*MAPK3*, *&* TNF by STRING database

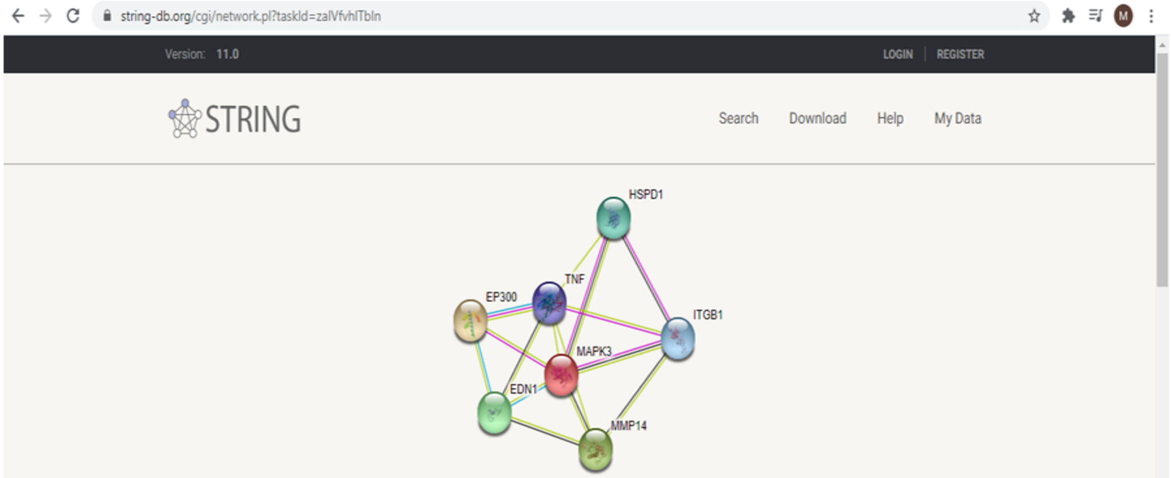

in cell proliferation gene and acute inflammatory resonse and cytokine signalling
